# Retinoic Acid and Retinoid X Receptors

**DOI:** 10.3390/cells12060864

**Published:** 2023-03-10

**Authors:** Michael Schubert, Pierre Germain

**Affiliations:** 1 Laboratoire de Biologie du Développement de Villefranche-sur-Mer, Institut de la Mer de Villefranche, Sorbonne Université, CNRS, 181 Chemin du Lazaret, 06230 Villefranche-sur-Mer, France; 2 Centre de Biologie Structurale, CNRS, INSERM, Univ Montpellier, 29 Rue de Navacelles, 34090 Montpellier, France

**Keywords:** nuclear receptor signaling, retinoic acid receptor, retinoid X receptor, pharmacology, structure/function relationships, genomic and non-genomic signaling, post-translational modification, homeostasis and disease, development, origin and evolution

One of the most fundamental discoveries in human biology was that of the existence of essential micronutrients that the body cannot synthesize but nonetheless requires for proper functioning [1]. However, decades passed between the moment of their discovery, the detailed description of their physiological roles, and the definition of their functions on cellular and molecular levels. A very good example for this historical progression is provided by vitamin A (which is all-*trans* retinol, a small, fat-soluble, organic compound) [2,3]. The molecule was first purified over 100 years ago, and it was shown soon thereafter to be essential for sustaining vision, reproduction, growth, immunity, and homeostasis as well as for promoting cell differentiation [4]. The function of vitamin A in vision was subsequently revealed to be carried out by 11-*cis* retinal and its association with opsin to form the chromophore rhodopsin [5]. Yet, the mechanisms underlying the other biological roles of vitamin A remained elusive.

Only several decades later, all-*trans* retinoic acid (RA) was identified as the biologically active metabolite of vitamin A, mediating the observable effects of vitamin A on cell growth and differentiation [6,7]. The intracellular receptor that transduces the signal conveyed by the presence of RA into a cellular response was finally discovered in 1987: the retinoic acid receptor (RAR or NR1B) [8,9]. The first identified RAR corresponded to human RARα (NR1B1), with the other two human RAR paralogs, RARβ and RARγ (NR1B2 and NR1B3, respectively), having been discovered only later. Since RARα, RARβ, and RARγ bind RA with similar affinity, following the identification and initial characterization of the receptors, specific synthetic ligands for each RAR paralog have been developed for pharmacological analyses and potential therapeutical applications [10].

Following the discovery of RAR, it was established that the receptor functions in a heterodimer with the retinoid X receptor (RXR or NR2B), binding directly to the regulatory DNA of target genes [11,12]. As for RAR, there are three RXR paralogs in humans, RXRα, RXRβ, and RXRγ. While all-*trans* RA exhibits no affinity for human RXRs, one of its isomers, 9-*cis* RA, can bind and activate all three RXRs. However, there is still no consensus regarding the existence of a natural, physiologically active ligand for these receptors. As for RAR, RXR-specific synthetic ligands have been generated, but none with apparent paralog selectivity, since the residues that constitute the ligand binding pockets of RXRα, RXRβ, and RXRγ are highly conserved [13].

Both RAR and RXR are members of a large superfamily of DNA binding proteins, called the nuclear hormone receptors (NRs) that are present in all multicellular animals [14,15]. In addition to RAR and RXR, members of this superfamily include receptors for thyroid hormones, steroids (estrogen, glucocorticoid, mineralocorticoid, progesterone, and androgen), and vitamin D, as well as several orphan receptors, whose endogenous ligands have not yet been identified [14,15]. While some NR superfamily members form heterodimers with RXR, in a similar manner to RAR, others act as homodimers or monomers [16]. Liganded NRs are ligand-dependent transcription factors, with ligand binding triggering conformational changes in the receptor that result in the recruitment of coregulators [14]. Some NRs, including the RXR-RAR heterodimer, have a dual functionality, acting as silencers of transcription in the absence of a ligand (due to their ability to recruit corepressors) and as activators of transcription in the presence of a ligand (due to their ability to recruit coactivators) [14]. In addition to this well-established genomic function in the nucleus, RA also exerts non-genomic effects, for example, by rapidly activating kinase cascades through a pool of RARs, localized either in the cytoplasm or at the plasma membrane [17]. Furthermore, both RAR and RXR are themselves regulated by phosphorylation, with several sites and associated kinases having already been identified [18].

The aim of this Special Issue is to present examples of currently ongoing research on RAR and RXR and, more generally, RA-dependent signaling to define the status quo and to highlight the impressive diversity in biological questions and experimental approaches. The collection, thus, covers topics as diverse as evolutionary origins, developmental roles, metabolic functions, and clinical implications of RA receptors and their ligands. As such, the 14 articles in this collection (9 research and 5 review papers), written by established experts in the field, reflect the complexity of contemporary research on RAR, RXR, and RA and define both the direction and challenges for future work on this intricate intercellular signaling system.

The article by Polvadore and Maden [19], for example, explores the roles of RAR during limb regeneration in the axolotl *Ambystoma mexicanum*, a salamander native to Mexico. Using a differential transcriptomic screening approach based on pharmacological treatments with synthetic RAR paralog-specific agonists, they identify RARα as the dominant RAR to control positional identity in the regenerating limb and define a list of candidates acting downstream of RARα during this process. RAR-dependent signaling is also at the center of the study by Abbou and colleagues [20]. The focus of this study, however, is on the evolutionary conservation of the RA metabolic and gene regulatory networks in the African clawed frog *Xenopus laevis*. Their results suggest that gene loss was an important factor in the evolution of the RA metabolic network, leading to tighter regulation and higher robustness of genetic responses. The article by Schmidt and colleagues [21] uses the concept of evolutionary comparisons and applies it to the development of the vertebrate pronephros. Their work makes use of three non-conventional model organisms: an invertebrate chordate (the cephalochordate amphioxus) and two vertebrates, a cyclostome (the lamprey) and a chondrichthyan (the catshark). They show that the anterior boundary of pronephric gene expression is conserved in the lamprey and the catshark and that RA signaling is involved in the regulation of this anterior boundary in the catshark. The situation in catsharks is, thus, similar to what has previously been described in amniote vertebrates.

The Special Issue further features articles exploring the role of RAR and RA signaling during spermatogenesis. Condrea and colleagues [22], for example, explore the role of RARα in spermatogonia and demonstrated, via cell-specific conditional mutagenesis, that RARα plays no crucial role in germ cells. Conversely, a temporally controlled, cell-specific mutagenesis approach provides crucial evidence for a requirement of RARα in Sertoli cells during puberty. A review article by Zhou and Wang [23] discusses the role of the blood–testis barrier in the RA-dependent regulation of spermatogenesis. The article summarizes previous work on the subject and exposes knowledge gaps that need to be addressed in the future by employing novel research methods, such as single-cell sequencing. In contrast, the review by Duester [24] focuses on the role of RA signaling in vertebrate eye development. The article discusses the requirement of RA for optic cup formation and morphogenesis of anterior eye structures and highlights the need for studies focusing on the identification of direct RA signaling targets during eye development. Yamakawa and Wada [25] use a comparative approach to review our current understanding of RA signaling in echinoderms, an important group of animals for studying the evolutionary diversification of RXR-RAR-dependent signaling. In addition to annotating RA signaling components in different echinoderms, they discuss possible roles for RA during echinoderm development and speculate on ancestral functions of this signaling system in echinoderms and beyond.

In their article, de Hoog and colleagues [26] describe the development of an assay system for assessing the transcriptional activity and ligand binding properties of RXRs from non-conventional invertebrate model organisms. Using cultures of central nervous systems dissected from larval fruit flies (*Drosophila melanogaster*), they set up a ligand sensor system to demonstrate that the RXR from the gastropod mollusk *Lymnaea stagnalis* is activated by different RA isomers. Unexpectedly, the sensor also detects endogenous RA-like activity in dissected central nervous systems of fruit fly larvae. The molecular interactions of RARs with their co-regulators are further explored in an article by Dahiya and colleagues [27]. They address the regulation of RARβ by the Sin3/MAD1 complex and provide evidence that this complex cooperates with classical corepressors, such as NCoR, in the regulation of RARβ. They hypothesize that the Sin3/MAD1 complex acts as a second repressor of RARβ, modulating its ligand sensitivity.

Chen [28] reviews the interactions of the vitamin A and insulin signaling systems in glucose and lipid metabolism. The article also provides a detailed account of the effects of vitamin A and RA on the metabolism and of the functional implications of RXR-RAR in these processes. Bhattacharya and colleagues [29] study epidermal homeostasis and, more specifically, the role of the transcriptional regulator BCL11A in this process. They find that ablation of Bcl11a in skin epidermal keratinocytes enhances cell proliferation and differentiation and promotes rapid closure of excisional wounds. BCL11A, thus, acts as a negative regulator of cutaneous wound healing. The epidermis-specific knockout created in this study will serve as a basis for future studies addressing the mechanistic basis of the accelerated healing phenotype, including the possible involvement of RXR-RAR. In their study, Yu and colleagues [30] address the interplay of retinol-binding protein type 1 (RBP1) expression and endogenous levels of RA. They find that both RBP1 and RA levels are reduced in mammary tumor tissue and tumorigenic epithelial cell lines and establish a direct link between RBP1 and RA levels that is maintained when RBP1 expression is restored therapeutically in cellular environments characterized by reduced RBP1 transcription.

The development of RAR agonists as therapeutic agents is explored in an article by Pignolo and Pacifici [31]. The review discusses chondrogenic cell differentiation and cartilage maturation during skeletogenesis, highlighting that these processes require the absence of RA-dependent signaling and active repression mediated by unliganded RARs. These findings provide the theoretical basis for the use of RAR agonists, and in particular of RARγ-specific agonists, in the treatment of heterotopic ossification, a pathological process defined by the formation of bone in soft tissues by recapitulating developmental skeletogenesis. The work by Shao and colleagues [32] focuses on the implication of RXR and another NR, the peroxisome proliferator-activated receptor (PPAR or NR1C), in breast cancer progression. Based on previous observations, they evaluate whether the cytoplasmic colocalization of RXRα and PPARγ can be used as a negative indicator for patient prognosis. Their results reveal a clear correlation between cytoplasmic coexpression of RXRα and PPARγ in breast cancer samples and a significantly shorter overall and disease-free survival, demonstrating that this coexpression is, indeed, an independent negative prognosticator for breast cancer patients. 

Taken together, the articles in this Special Issue offer a glimpse into both the state of the art and the diversity of research on RAR, RXR, and RA signaling, from developmental roles to reproduction and evolution, from molecular interactions to metabolic disorders, disease, and cancer. The topics covered are as diverse as the methodological approaches are original. One unifying feature of the contributions is that they reveal perspectives for future work on the subject, hence, setting the stage for novel, exciting discoveries in the years to come. Technological advances and the availability of an ever-expanding number of animal systems will continue to boost scientific research and, thus, our efforts to understand the complexity of the molecular mechanisms controlled by RAR and RXR and how they translate into physiological outputs. We hope that the articles in this Special Issue will serve as a source of inspiration for reflections and discussions into the scope and direction of scientific projects shaping the future of retinoid receptor research.

## References

[1] Drummond J.C. (1920). The nomenclature of the so-called accessory food factors (vitamins). Biochem. J..

[2] Giguère V., Evans R.M. (2022). Chronicle of a discovery: The retinoic acid receptor. J. Mol. Endocrinol..

[3] Petkovich M., Chambon P. (2022). Retinoic acid receptors at 35 years. J. Mol. Endocrinol..

[4] Semba R.D. (2012). On the ‘discovery’ of vitamin A. Ann. Nutr. Metab..

[5] Wald G. (1935). Carotenoids and the visual cycle. J. Gen. Physiol..

[6] Ito Y.L., Zile M., Ahrens H., DeLuca H.F. (1974). Liquid-gel partition chromatography of vitamin A compounds; formation of retinoic acid from retinyl acetate in vivo. J. Lipid Res..

[7] Strickland S., Mahdavi V. (1978). The induction of differentiation in teratocarcinoma stem cells by retinoic acid. Cell.

[8] Giguère V., Ong E.S., Segui P., Evans R.M. (1987). Identification of a receptor for the morphogen retinoic acid. Nature.

[9] Petkovich M., Brand N.J., Krust A., Chambon P. (1987). A human retinoic acid receptor which belongs to the family of nuclear receptors. Nature.

[10] Le Maire A., Teyssier C., Balaguer P., Bourguet W., Germain P. (2019). Regulation of RXR-RAR heterodimers by RXR- and RAR-specific ligands and their combinations. Cells.

[11] Leid M., Kastner P., Lyons R., Nakshatri H., Saunders M., Zacharewski T., Chen J.Y., Staub A., Garnier J.M., Mader S. (1992). Purification, cloning, and RXR identity of the HeLa cell factor with which RAR or TR heterodimerizes to bind target sequences efficiently. Cell.

[12] Germain P., Rochel N., Bourguet W. (2021). Ligands and DNA in the allosteric control of retinoid receptors function. Essays Biochem..

[13] Le Maire A., Rey M., Vivat V., Guée L., Blanc P., Malosse C., Chamot-Rooke J., Germain P., Bourguet W. (2022). Design and *in vitro* characterization of RXR variants as tools to investigate the biological role of endogenous rexinoids. J. Mol. Endocrinol..

[14] Germain P., Chambon P., Eichele G., Evans R.M., Lazar M.A., Leid M., De Lera A.R., Lotan R., Mangelsdorf D.J., Gronemeyer H. (2006). International Union of Pharmacology. LX. Retinoic acid receptors. Pharmacol. Rev..

[15] Miglioli A., Canesi L., Gomes I.D.L., Schubert M., Dumollard R. (2021). Nuclear receptors and development of marine invertebrates. Genes.

[16] Beinsteiner B., Markov G.V., Bourguet M., McEwen A.G., Erb S., Patel A.K.M., El Khaloufi El Khaddar F.Z., Lecroisey C., Holzer G., Essabri K. (2022). A novel nuclear receptor subfamily enlightens the origin of heterodimerization. BMC Biol..

[17] Al Tanoury Z., Piskunov A., Rochette-Egly C. (2013). Vitamin A and retinoid signaling: Genomic and nongenomic effects. J. Lipid Res..

[18] Rochette-Egly C. (2015). Retinoic acid signaling and mouse embryonic stem cell differentiation: Cross talk between genomic and non-genomic effects of RA. Biochim. Biophys. Acta.

[19] Polvadore T., Maden M. (2021). Retinoic acid receptors and the control of positional information in the regenerating axolotl limb. Cells.

[20] Abbou T., Bendelac-Kapon L., Sebag A., Fainsod A. (2022). Enhanced loss of retinoic acid network genes in *Xenopus laevis* achieves a tighter signal regulation. Cells.

[21] Schmidt P., Leman E., Lagadec R., Schubert M., Mazan S., Reshef R. (2022). Evolutionary transition in the regulation of vertebrate pronephros development: A new role for retinoic acid. Cells.

[22] Condrea D., Souali-Crespo S., Féret B., Klopfenstein M., Faisan S., Mark M., Ghyselinck N.B., Vernet N. (2022). Retinoic acid receptor alpha is essential in postnatal Sertoli cells but not in germ cells. Cells.

[23] Zhou Y., Wang Y. (2022). Action and interaction between retinoic acid signaling and blood-testis barrier function in the spermatogenesis cycle. Cells.

[24] Duester G. (2022). Towards a better vision of retinoic acid signaling during eye development. Cells.

[25] Yamakawa S., Wada H. (2022). Machinery and developmental role of retinoic acid signaling in echinoderms. Cells.

[26] De Hoog E., Saba Echezarreta V.E., Turgambayeva A., Foran G., Megaly M., Necakov A., Spencer G.E. (2022). Molluscan RXR transcriptional regulation by retinoids in a *Drosophila* CNS organ culture system. Cells.

[27] Dahiya N.R., Leibovitch B.A., Kadamb R., Bansal N., Waxman S. (2022). The Sin3A/MAD1 complex, through its PAH2 domain, acts as a second repressor of retinoic acid receptor beta expression in breast cancer cells. Cells.

[28] Chen G. (2021). The interactions of insulin and vitamin A signaling systems for the regulation of hepatic glucose and lipid metabolism. Cells.

[29] Bhattacharya N., Indra A.K., Ganguli-Indra G. (2022). Selective ablation of BCL11A in epidermal keratinocytes alters skin homeostasis and accelerates excisional wound healing in vivo. Cells.

[30] Yu J., Perri M., Jones J.W., Pierzchalski K., Ceaicovscaia N., Cione E., Kane M.A. (2022). Altered RBP1 gene expression impacts epithelial cell retinoic acid, proliferation, and microenvironment. Cells.

[31] Pignolo R.J., Pacifici M. (2021). Retinoid agonists in the targeting of heterotopic ossification. Cells.

[32] Shao W., Köpke M.B., Vilsmaier T., Zati Zehni A., Kessler M., Sixou S., Schneider M., Ditsch N., Cavaillès V., Jeschke U. (2022). Cytoplasmic colocalization of RXRα and PPARγ as an independent negative prognosticator for breast cancer patients. Cells.

